# Different surgical approaches in laparoscopic sleeve gastrectomy and their influence on metabolic syndrome: A retrospective study

**DOI:** 10.1097/MD.0000000000009699

**Published:** 2018-01-26

**Authors:** Hady Razak Hady, Magdalena Olszewska, Mikolaj Czerniawski, Dawid Groth, Inna Diemieszczyk, Patrycja Pawluszewicz, Adam Kretowski, Jerzy Robert Ladny, Jacek Dadan

**Affiliations:** a 1st Department of General and Endocrinological Surgery; b Department of Endocrinology, Diabetology and Internal Medicine; c Department of Emergency Medicine and Disasters, Medical University of Bialystok, Bialystok, Podlaskie, Poland.

**Keywords:** bariatric surgery, bougie size, laparoscopic sleeve gastrectomy, metabolic syndrome

## Abstract

Obesity is a growing health, social, and economic issue and became an epidemic, according to recent report of World Health Organization.

The only method with scientifically proved efficiency of body mass loss is a surgical treatment. Laparoscopic sleeve gastrectomy (LSG) is recently a leading method in metabolic surgery. There are no standards of operative technique for LSG so far. The influence of technique modification on metabolic effect has not been described clearly.

The aim of this study was to evaluate metabolic effects in patients with morbid obesity who underwent various surgical approaches of LSG.

The study included 120 patients who were randomly divided into 3 groups: Group I, where bougie size was 32 French (Fr), Group II—36 Fr and Group III—40 Fr. Each group was divided into 2 subgroups, based on the distance of resection beginning from the pylorus—2 or 6 cm. Statistical analysis of: body mass index (BMI), the Percentage of Excess Weight Loss (%EWL), the Percentage of Excess BMI Loss (%EBMIL), levels of glucose and insulin on an empty stomach, glycated hemoglobin (HbA1c), insulin resistance (Homeostatic Model Assessment of Insulin Resistance Index—HOMA-IR), aspartate transaminase (AST), alanine transaminase (ALT), total cholesterol, high-density lipoprotein (HDL), low-density lipoprotein (LDL), triglycerides (TG), and C-reactive protein (CRP) were under investigation.

Statistically significant decrease in body mass, BMI, %EWL, %EBL, glucose, and insulin concentrations has been observed in all studied groups. It was the highest when the smallest calibration tube has been used (32 Fr). Similar results were observed in HOMA-IR and HbA1c levels. Statistically significant decrease of total cholesterol, LDL, and TG concentrations have been observed. Significant increase of HDL in every group has been also noted. Postoperative CRP values were the lowest when the smallest bougie was used.

LSG is effective method of obesity treatment. Metabolic effects of LSG are the most noticeable when a small bougie size is used.

## 1 Introduction

Obesity is a process of excessive fat accumulation in the body which results in homeostasis breakdown and causes biochemical and physiological dysfunctions of tissues. Obesity also leads to the development of comorbidities such as hypertension, diabetes mellitus type 2, sclerosis, nonalcoholic fatty liver disease (NAFLD), goat, obstructive sleep apnea, and tumors.^[1–4]^

Conservative treatment, which consists of diet, lifestyle modification, cognitive behavioral therapy, and pharmacotherapy, must be systematic and long-term. However, body weight loss success rate does not exceed 10%.^[5]^ Due to unsatisfactory results of aforementioned methods, surgical treatment has become increasingly important. Obesity surgery is the most effective way to long-term weight loss.^[6]^ Nowadays, among many surgical approaches, laparoscopic sleeve gastrectomy (LSG) gains popularity, because of satisfactory weight loss and remission of comorbidities. Final effect of the therapy is a result of decreased stomach volume and, in consequence, lower food intake. Moreover, recent studies suggest that resecting a larger part of the stomach (fundus and body), affects gastrointestinal tract peristalsis, neurohormonal system, and carbohydrate–fat balance.^[7,8]^ LSG has no established standards regarding the diameter of the stomach left after the surgery. Recommendations of surgical associations are a result of clinical reports and metaanalyses, however, there is still a lack of final agreement on the technique of LSG. The knowledge about the influence of surgical approach on patient metabolic response is still incomplete.

The aim of this study was to analyze body weight, BMI, the Percentage of Excess Weight Loss (%EWL), the Percentage of Excess BMI Loss (%EBMIL) in patients with morbid obesity who underwent various surgical approaches of LSG: different diameter of bougie for stomach volume calibration and different distance of cut-off line from the pylorus. Levels of glucose and insulin on an empty stomach, glycated hemoglobin (HbA1c), insulin resistance (Homeostatic Model Assessment of Insulin Resistance Index—HOMA-IR), aspartate transaminase (AST), alanine transaminase (ALT), total cholesterol, high-density lipoprotein (HDL), low-density lipoprotein (LDL), triglycerides (TG), and C-reactive protein (CRP) were also under investigation.

## 2 Patients and methods

The material consists of 120 patients hospitalized in 1st Department of General and Endocrinological Surgery, Medical University of Bialystok, between 2012 and 2014 who underwent LSG in order to treat morbid obesity. Patients were divided into 3 groups by the bougie size: Group I, where bougie size was 32 French (Fr), Group II—36 Fr and Group III—40 Fr. The bougie size was chosen randomly for each patient before the surgery. Each group was divided into 2 subgroups, based on the distance of resection beginning from the pylorus—2 or 6 cm.

Criteria of qualification of patients to the surgery were described previously.^[9]^ All patients had met at least 3 criteria necessary for the diagnosis of metabolic syndrome according to the International Diabetes Federation.^[10]^ Follow-up of the level of metabolic syndrome reduction was limited to 1 year.

All patients provided written informed consent before the study and additional written informed consent was obtained before the surgical procedure. This study was approved by the Ethics Committee of the Medical University of Bialystok, Poland (No R-I-002/438/2014) in accordance with the guidelines of the Helsinki Declaration.

There were 76 female (63.3%) and 44 male (36.7%) patients in examined group. Average age was 43. Groups characteristics are shown in Table 1.

**Table 1:**
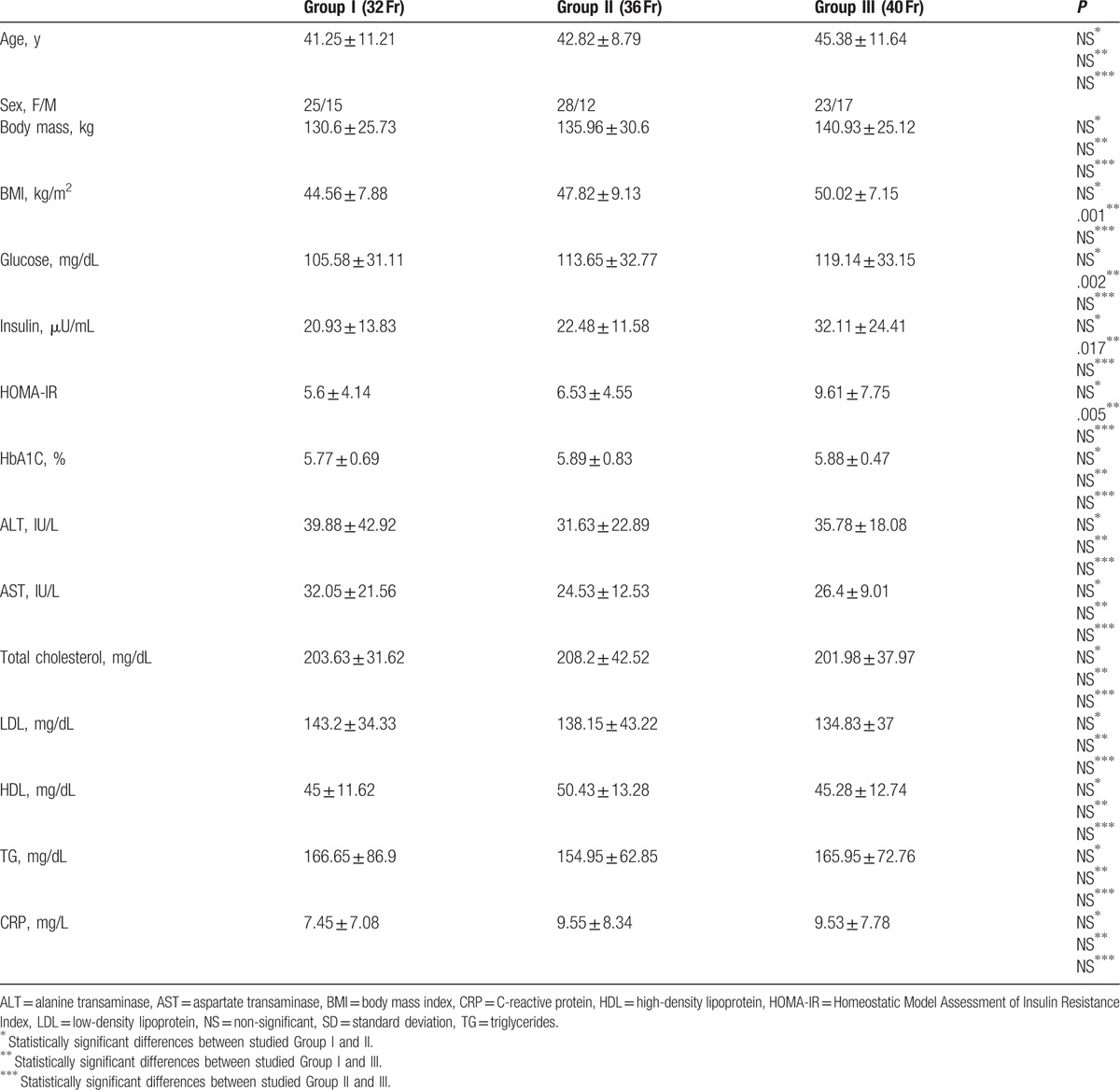
Patients characteristics before surgery (mean and SD).

One team of surgeons (1 operator and 2 assistants) performed all surgeries, according to the procedure described before.^[9]^ Cut-off line of the omentum reached upwards the left diaphragmatic branch and downwards approximately 2 or 6 cm from the pylorus. The stomach was reduced to the bougie size 32, 36, or 40 Fr (Group I, Group II, and Group III). Leak test was performed with 5% glucose and air insufflation. Patients were discharged home in the second or third day after the surgery and were regularly examined by clinical dietician and surgeon during the follow-up period.

All patients were examined 1, 3, and 6 months after the surgery. Fasting 10 to 12 hours blood was taken for a clot tube and then centrifuged until serum was obtained. Insulin, glucose, total cholesterol, HDL, LDL, and TG were evaluated in order to control changes in particular time points after the surgery. Rates of %EWL, %EBMIL, and HOMA-IR were calculated according to the following formulas:

(1) %EWL = (preoperative weight − follow-up weight)/(preoperative weight − ideal weight) × 100
  For ideal weight calculations, the Lorenz formulas were used:
  Ideal female weight = (height in cm − 100) − ((height in cm − 150)/2),
  Ideal male weight = (height in cm − 100) − ((height cm − 150)/4).
(2) %EBMIL = (preoperative BMI − follow-up BMI)/(preoperative BMI − 25) × 100
(3) HOMA-IR = glucose level (mg/dL) × insulin concentration (mU/L)/405; quotient >2.6 supported insulin resistance.

All data were extracted from original sources to fields within an Excel (Microsoft, Redmond, WA) database. Data manipulation and analysis was conducted using SPSS statistical software for Windows, version 21 (IBM SPSS, Chicago, IL). Selected demographic (age, weight, body mass index [BMI, kg/m^2^]) and surgical technique (bougie size and distance from pylorus) variables were estimated using mean, SD, range, and the percentage of studies reporting on each variable.

Comparison of 3 studied groups was performed using ANOVA with post hoc Tukey test. Pairwise *t* test was used to evaluate the statistical significance between the same groups in the different periods of the follow-up (1, 3, and 6 months after the surgery). Pearson test was used to determine whether there were any differences in the distribution of gender by reinforcement method.

Statistical tests were 2-tailed and values of *P* < .05 were considered statistically significant.

Pearson test was used to determine whether there were any correlations between studied groups. Values of *P* < .05 were considered statistically significant. All calculations were performed by professional statistician.

## 3 Results

In all studied groups a statistically significant decrease in body mass and BMI was observed. The highest weight loss was observed in Group I, where the smallest bougie was used (32 Fr) compared to Group II and Group III (Table 2).

**Table 2:**
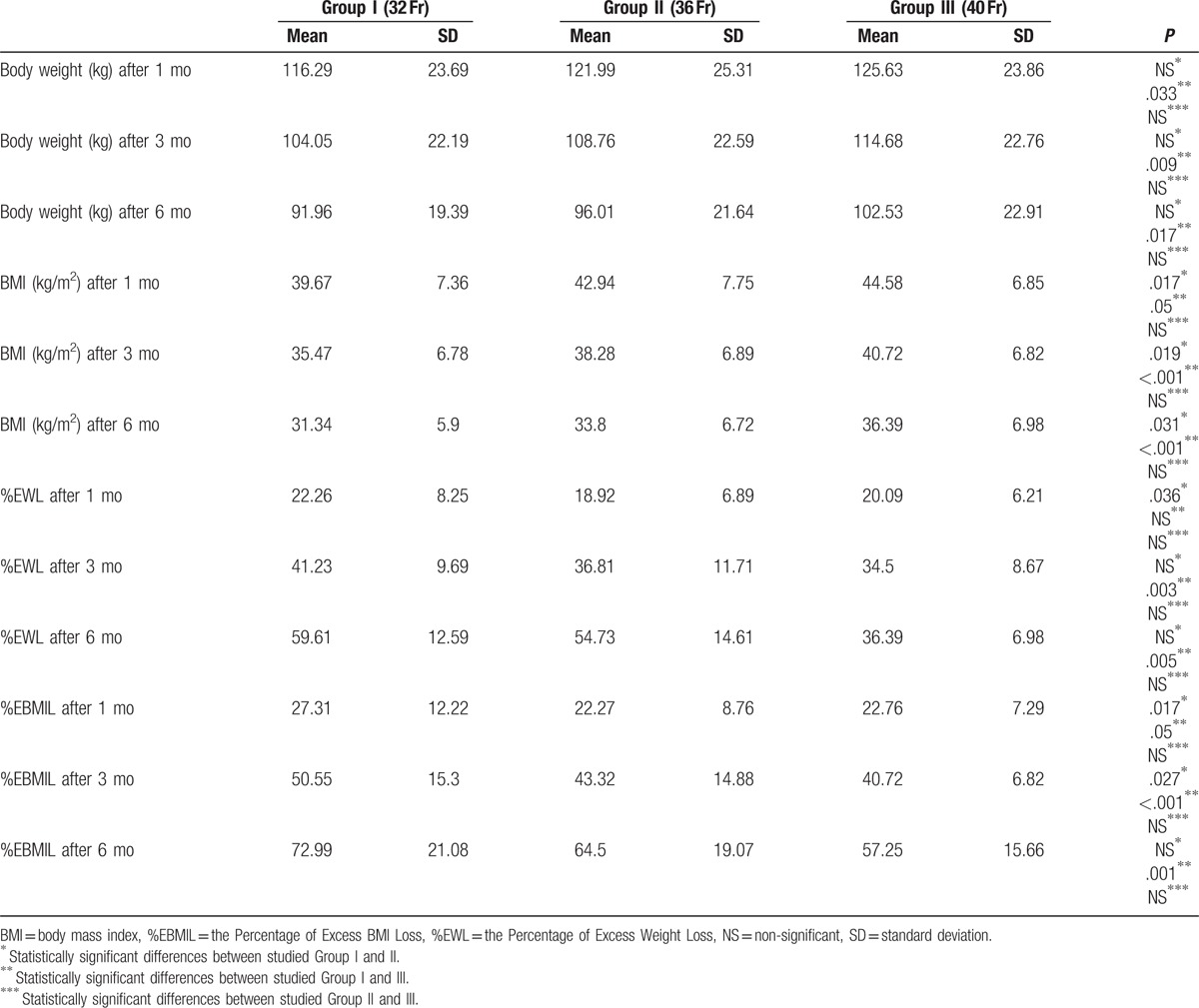
Postoperative changes in body weight, BMI, %EWL and %EBMIL.

**Figure 1:**
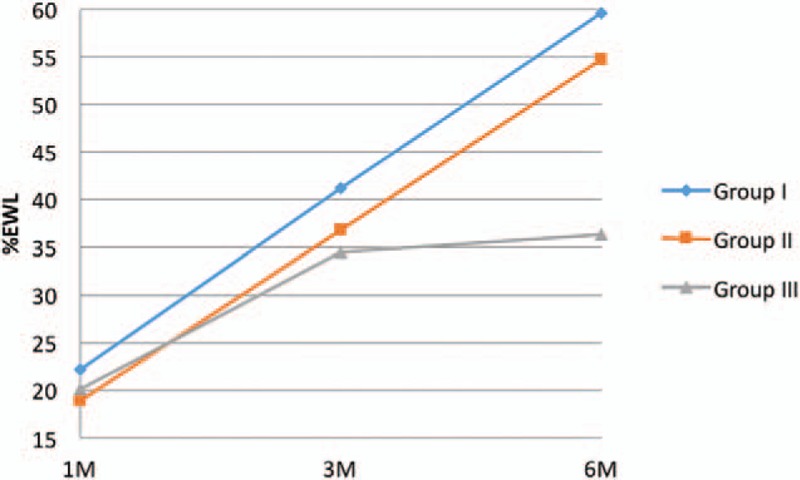
Changes of %EWL 1, 3, and 6 months after the surgery.

Postoperative dynamics of weight and BMI loss were evaluated using %EWL and %EBMIL. During 6 months follow-up period, the most noticeable decrease of body weight and BMI was observed in Group I. One month after the surgery, %EWL in Group I was 22.26% ± 8.25%, after 3 months was equal to 41.23 ± 9.69%, and after 6 months reached 59.61 ± 12.59%; the decrease was statistically significant (Table 2, Fig. 1).

**Figure 2:**
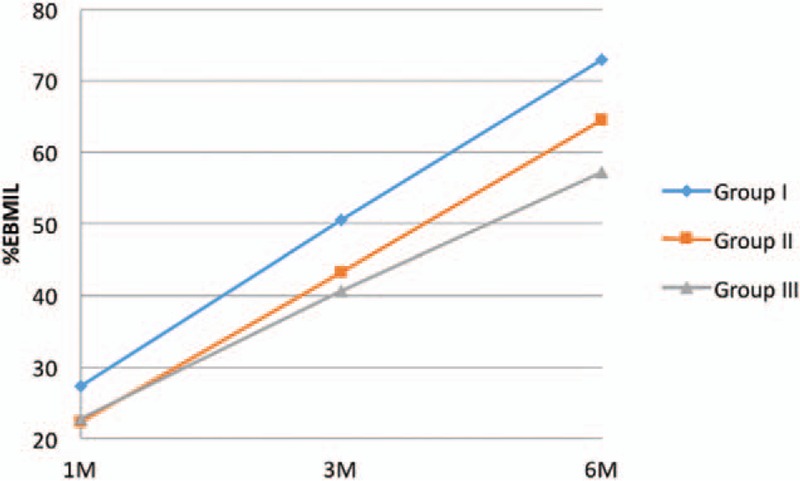
Changes of %EBMIL 1, 3, and 6 months following the LSG.

BMI loss measured with %EBMIL was significantly lower in all studied groups. One month after the surgery in Group I it was equal to 27.31 ± 12.22%. In 3 months follow-up, it was 50.55 ± 15.3% and at the end of the observation period reached 72.99 ± 21.08% (Table 2, Fig. 2). There were no statistically significant differences between 2 and 6 cm cut-off lines start from the pylorus.

In our research, a glucose level was also measured and compared with preoperative results. During 6 months follow-up, statistically significant changes in glucose concentration in plasma have been observed in every stage of observation and in all studied groups (Table 3). The highest decrease was observed in Group I and II (Table 3).

**Table 3:**
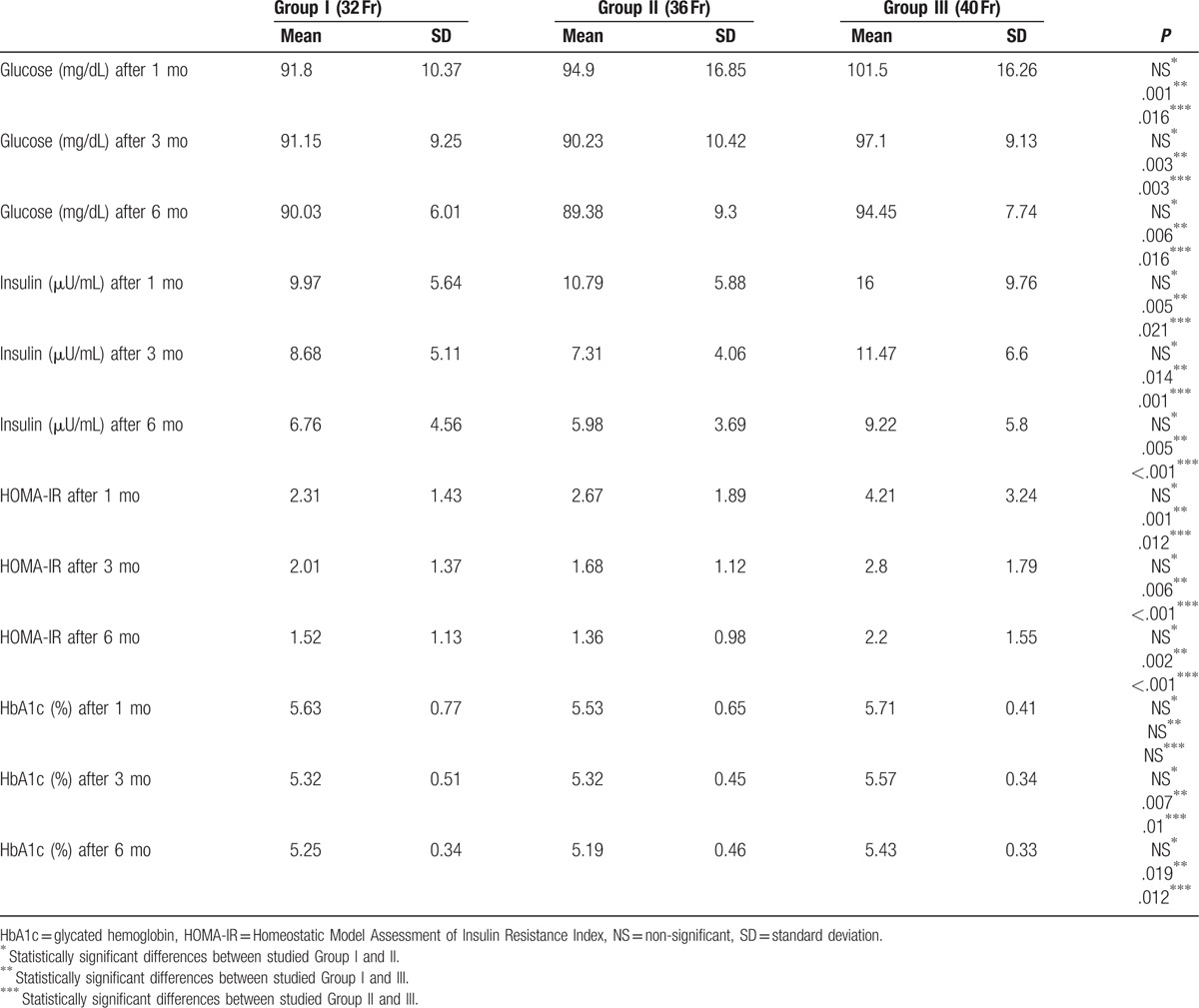
Postoperative changes in carbohydrate metabolism.

All measurements of insulin concentration were also statistically significant with a tendency to decrease in comparison to preoperative values (Table 3). The greatest reduction in concentration of insulin in serum was observed 1 month after the surgery. Three and 6 months postoperatively, the values also decreased, but less dynamically. However, the results were statistically significant in every stage of the study. Resection 2 cm from the pylorus resulted in more dynamic decrease of insulin concentration than 6 cm approach—1 month after the surgery in Group I (respectively, 8.415 and 11.53 μU/mL) and 3 months after the treatment in Group II (5.842 and 8.785 μU/mL).

**Figure 3:**
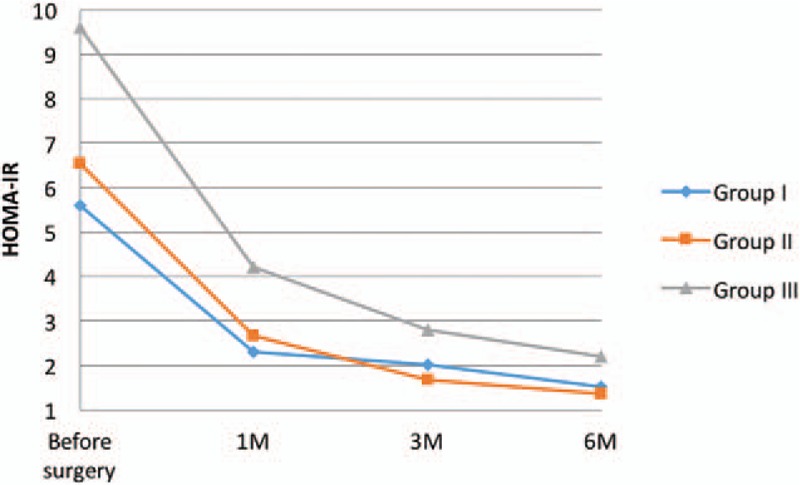
Changes of HOMA-IR 1, 3, and 6 months after the surgery.

Insulin and glucose concentrations allowed us to measure HOMA-IR. Reduction of insulin resistance was discovered in every studied group, however in Group I it was the most noticeable (2.31 ± 1.43; Table 3, Fig. 3). Value of HOMA-IR was statistically significant in every stage of observation. Moreover, a 2 cm cut-off line from the pylorus approach was related to lower HOMA-IR 1 month after the LSG in Group I (1.907 compared with 2.718; *P* = .007) and 3 months after the surgery in Group II (1.248 vs 2.109; *P* = .018).

**Figure 4:**
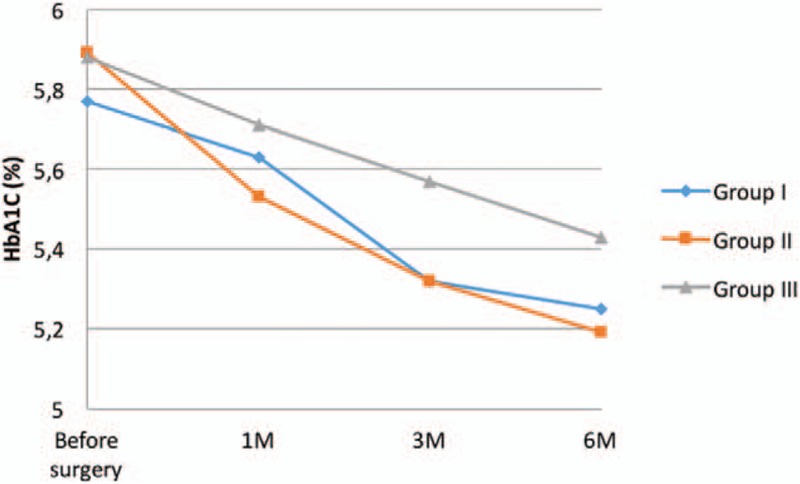
Changes of HbA1c 1, 3, and 6 months after the surgery.

Our study revealed statistically significant decrease in HbA1c in studied Group I and II, 3 months after the surgery (5.32 ± 0.51% and 5.32 ± 0.45%) and 6 months after the surgical treatment (5.25 ± 0.34 and 5.19 ± 0.46) (Table 3, Fig. 4). Additionally, high correlation between weight (R = 0.52; *P* = .0006) and BMI (R = 0.46; *P* = .0026) before and 6 months after the surgery (R = 0.42; *P* = .007 vs R = 0.34; *P* = .034) and HbA1c was observed. In Group II weight and BMI correlated with HbA1c only before the surgery (R = 0.47; *P* = .002). In Group III the relationship was not calculated. However, in this study Group, the 2 cm starting line from the pylorus resulted in statistically significant higher HbA1c level compared to the 6 cm approach (3 months after the surgery, 5.69% and 5.2%; *P* = .022). In 6 months follow-up, the values were 5.58% (2 cm from the pylorus) and 5.28% (6 cm); *P* = .004.

**Figure 5:**
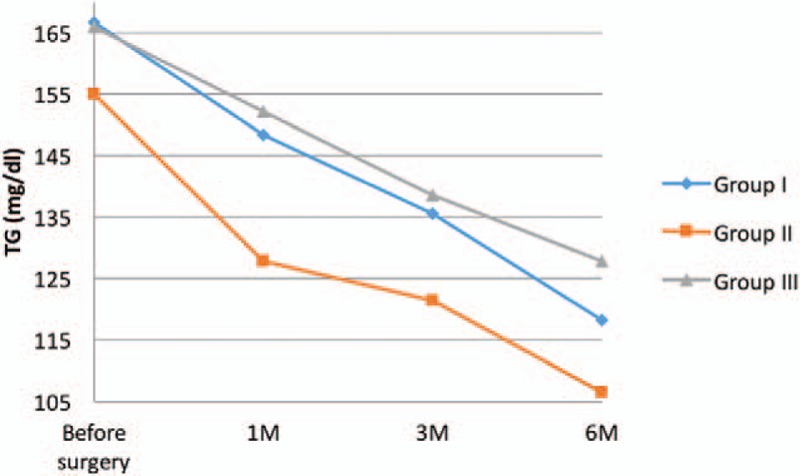
Changes of triglycerides 1, 3, and 6 months after the LSG.

Our study did not show any abnormalities in AST and ALT levels in obese patients. We observed that ALT concentration significantly decreased 6 months after the LSG in all studied groups (Table 4, Fig. 5).

**Table 4:**
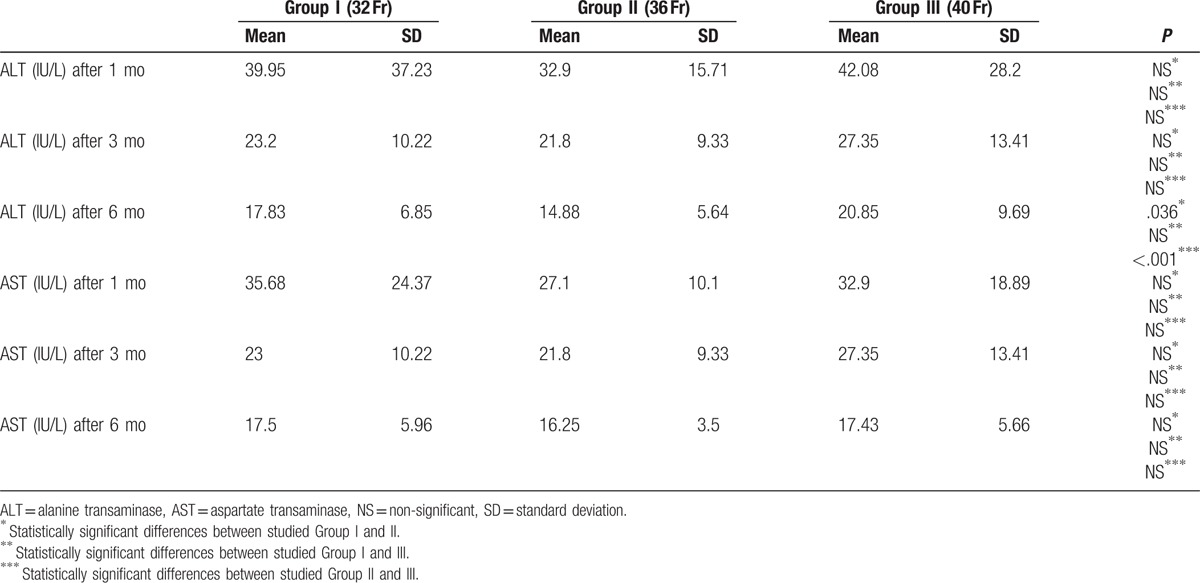
Postoperative changes in transaminases.

We have also studied the effect of LSG on lipid profile of obese patients. During the follow-up, statistically significant decrease in total cholesterol level in all studied group was observed, however, the reduction was the most dynamic in Group I (Table 5). Analyzing the differences in surgical approach, patients after 2 cm resection from the pylorus reached statistically lower levels of total cholesterol than patients in whom the resection started 6 cm from the pylorus (177.7 mg/dL vs 192.7 mg/dL; *P* = .028).

**Table 5:**
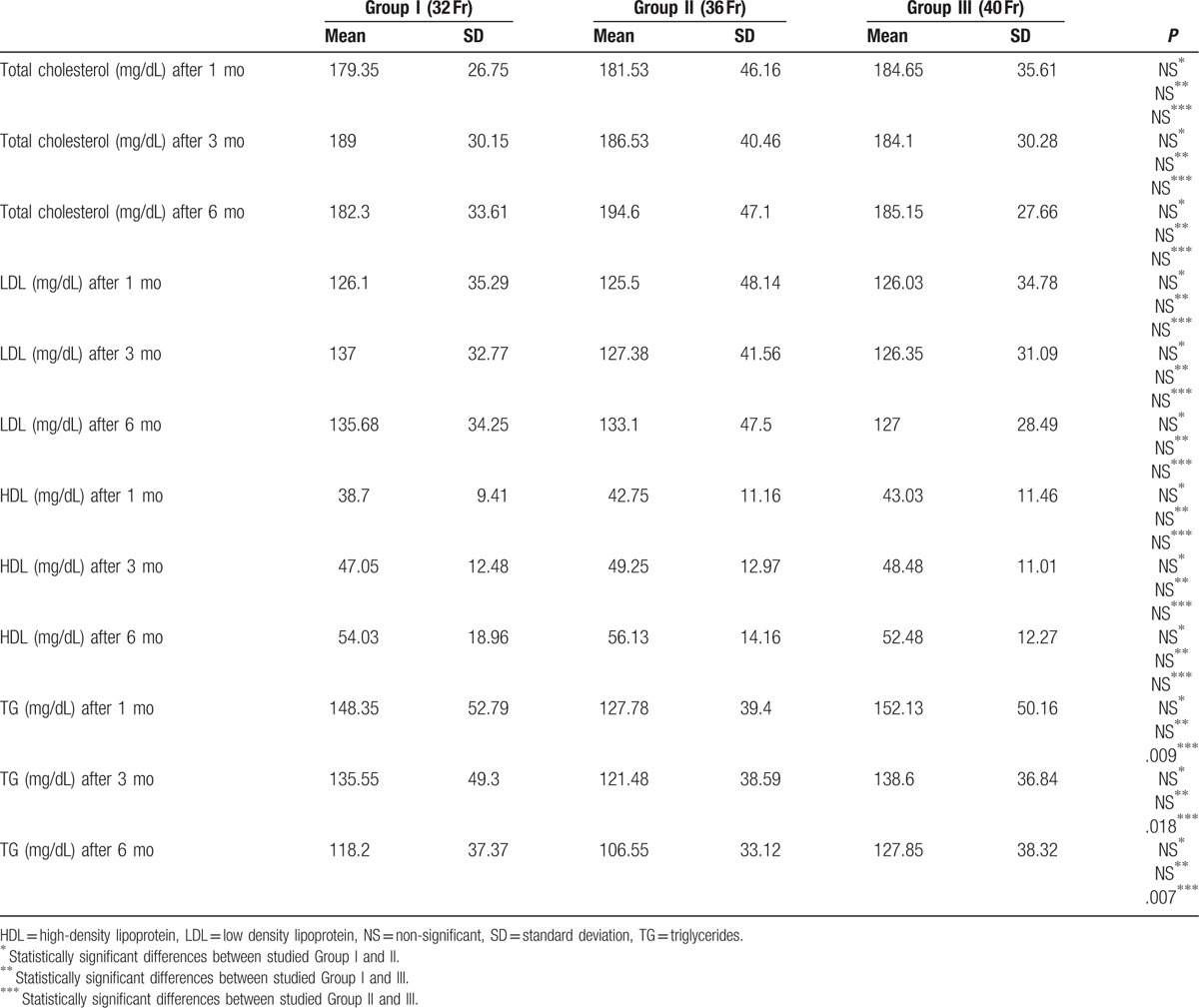
Postoperative changes in lipid profile parameters.

Only at the beginning of the follow-up period the reduction of LDL was statistically significant in all studied groups (126.1 ± 36.29 mg/dL vs 125.5 ± 48.14 mg/dL vs 126.03 ± 34.78 mg/dL) compared to the preoperative values (Table 5).

Changes in HDL level occurred 3 months after the LSG and it raised in all studied groups (data not statistically significant), however, 6 months after the surgery, HDL values significantly increased in every group (Table 5). In patients who underwent LSG with 2 cm cut-off line from the pylorus, where the bougie size was 40 Fr (Group III), a significant increase in HDL, 3 and 6 months after the surgery, was observed in comparison to the patients after 6 cm resection (after 3 months—52.7 mg/dL vs 44.25 mg/dL; *P* = .015 and 6 months—58.05 mg/dL vs 46.9 mg/dL; *P* = .002).

We have stated a statistically significant decrease in TG in all studied groups, 3 and 6 months postoperatively (Table 5).

Our study also included an evaluation of CRP concentration in plasma. Average level was between reference ranges in all studied groups during the whole follow-up, however, CRP values in Group I were statistically lower in comparison to Group III in every stage of the study (Table 6).

**Table 6:**
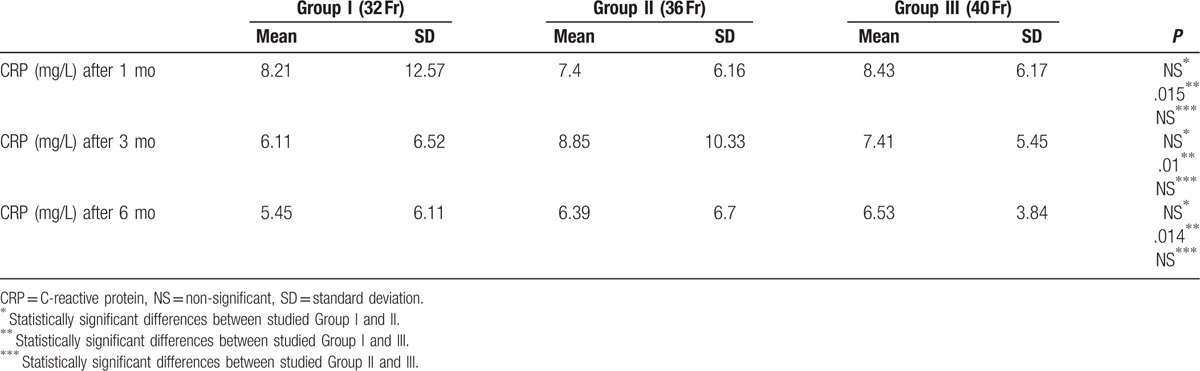
Postoperative changes in C-reactive protein.

The complication rate in our study group was 2.5% (n = 3). The complications were: acute pancreatitis (0.83%), bleeding from the first trocar site into the abdominal cavity (0.83%) and superficial thrombophlebitis of the lower extremity (0.83%).

## 4 Discussion

LSG has many advantages such as simplicity of the technique and short operative time (short anesthesia and less postoperative complications). LSG approach does not need any bypass anastomosis, so the physiological passage of gastrointestinal tract is not interrupted.

Our study reveals that the highest BMI and body weight reduction occurs when the smallest bougie size is used (32 Fr vs 36 Fr vs 40 Fr). The decrease was significantly higher in studied Group I compared with Group III. Six months after the surgery in studied Group I patients lost 59.61% of their preoperative weight and 72.99% of BMI (measured with %EWL and %EBMIL). Langer et al obtained similar results.^[8]^ In their study, %EWL in 6 months follow-up was 61.4% ± 16.3, although the bougie size was 48 Fr. Parikh et al^[11]^ observed that differences in %EWL were around 40% between studied groups (40 Fr vs 60 Fr), however, the results were not statistically significant. It is worth mentioning, that bougie sizes more than 40 Fr are rarely used. Yuval et al^[12]^ compared two LSG approaches with different bougie sizes (<40 Fr vs ≥40 Fr) and stated no statistically significant differences in %EWL between the groups.

Unsatisfactory weight loss forces surgeons to change the surgical technique for more restrictive.^[13]^ There is still lack of evidence that smaller bougie size is related to more intensive weight loss. Mongol et al^[14]^ reach %EWL around 41% with bougie size 32 Fr, while Han et al^[15]^ observe 72% of weight loss with bougie size 48 Fr.

It is a well-known fact that dysfunction of carbohydrates system, diabetes mellitus inclusive, is highly related with obesity and lack of physical activity.^[16]^ Ninety percent of diabetes mellitus type 2 patients are obese or overweight.^[17]^ We have found that LSG improves glycemic profile, insulin concentration, and HbA1c level, even before significant weight reduction. We have observed a gradual reduction in glucose concentration in each studied group. HbA1c level was also decreased in every group, most noticeably in Group I and II, 3 months after the surgery. The most significant reduction of insulin was observed 1 month after the treatment in Group I. In further follow-up a statistically significant decrease was discovered, but not as dynamic as at the beginning. Similar changes in HOMA-IR were observed. We stated that the most noticeable reduction of insulin resistance was in 1 month after the LSG, and then it normalized. Rizzello et al observed HOMA-IR decrease in third postoperative day. Two weeks after the surgery he found that glucose, insulin, and HOMA-IR values were significantly lower than before the surgery and occurred before the noticeable weight loss.^[18]^ Similar results published Catoi et al^[19]^ where 7 days after the LSG insulin resistance decrease was observed and it reached a statistically significant reduction at 30th day of the follow-up. Sharma et al studied a case of 49 years old male (BMI = 59 kg/m^2^) who after a LSG achieves a rapid (14 days) insulin concentration decrease, moreover HOMA-IR reaches 4.6 compared with 18.82 preoperatively.

Improvement of carbohydrates system after the LSG is highly related with weight and fat tissue loss. Recent studies show that changes in metabolism of carbohydrates occur few days after the LSG.^[20]^ We believe that it is caused by neurohormonal changes of digestive tract. Resection of major part of the stomach results in removal of cells which produce ghrelin (mainly in fundus). According to different studies, ghrelin level decreases about 40% to 50% in comparison with preoperative values.^[20–23]^ Reduction of ghrelin concentration decreases appetite, lowers glucose level, increases insulin secretion, and improves insulin resistance.

Additional mechanism which explains process of better carbohydrates metabolism is regulation of incretin hormones. Influence of bariatric procedures on normalization of glucose level is explained by hindgut hypothesis, which holds that digestive system contents have faster contact with the distant intestine and it leads to increased GLP-1 (glucagon-like peptide 1) and PYY (protein YY) secretion. Physiology of this process was studied in RYGB (Roux-Y gastric bypass), however, in LSG it is still unclear.^[8,24,25]^ It is hypothesized that LSG results in faster stomach emptying and rapid passage of not fully digested food through duodenum and proximal intestine.^[26–28]^

Moreover, in postoperative period, a lower secretion of hydrochloric acid in stomach is observed, which directly enhances production of PYY and secretion of gastrin and GLP-1. Karamanakos et al^[23]^ prove that after LSG, a PYY level increases and ghrelin secretion is reduced. Basso et al^[29]^ observed a GLP-1 and PYY increase in early postoperative period which confirms the results of Peterli et al.^[30]^ Rise of GLP-1 and PYY is responsible for lower appetite, glucose level reduction, insulin resistance restoration, inhibition of glucagon secretion, and in consequence, inhibition of gluconeogenesis. These findings explain different results between patients who underwent a stomach resection 2 and 6 cm from the pylorus. The 2 cm starting line from the pylorus resulted in faster passage of not fully digested food than in resection which started 6 cm from the pylorus. Therefore, an insulin secretion, HOMA-IR and total cholesterol level were lower in 2 cm approach. Surprisingly, HbA1c level was significantly higher in Group III (40 Fr), when resection started 2 cm from the pylorus. This might be explained by resection of the different number of cells which produce ghrelin, but also by the negative correlation between BMI and ghrelin level.^[31]^ Group III was the only one, where correlation between BMI and HbA1c was not observed, moreover BMI in this group was higher than in Group I and II, thus the production of ghrelin and influence on carbohydrates metabolism was lower.

Different studies show that steatohepatitis accompanies 60% of obese adults and 55% of children.^[32,33]^ Standard abdominal ultrasound is characterized by low sensitivity and specificity in detecting a steatohepatitis.^[34–36]^ Thus, in our study, we have observed concentrations of AST and ALT in the blood. In every step of the study the liver enzymes were between laboratory reference range values. We did not find any statistically significant differences in AST and ALT levels between the studied groups.

Obesity is connected with defects in metabolism of lipids, which result in higher risk of development of cardiovascular diseases.^[37]^ In our research, LSG improved all studied lipid parameters. Total cholesterol, LDL, and TG values significantly decreased in all groups, but LDL values did not reach the laboratory reference range at the end of the study.

Vix et al^[38]^ present similar results. They obtained a short-term reduction of total cholesterol and LDL after the LSG, however, at the end of their study, the values were even higher than in the preoperative period. Iannelli et al^[39]^ indicate statistically insignificant increase of total cholesterol and LDL 6 months after the LSG. On the other hand, Zhang et al^[40]^ did not observe any changes in concentration of total cholesterol and LDL in patients who underwent LSG. It is hypothesized that normal concentration of total cholesterol in obese patients might be a consequence of changes in expression of the receptors, which are responsible for lipids absorption. It may be also caused by changes in gastrointestinal microbiota or viral infection.^[41,42]^

Important, but less known, is influence of leptin on gluconeogenesis and lipolysis in fat tissue. Obesity is a state of elevated concentration of leptin in the blood, at the same time, it is related to tissue resistance to leptin.^[43]^

In our study, HDL concentration significantly increased during the follow-up, however, results before the third postoperative month were unsatisfactory. Zhang et al^[40]^ and Wong et al^[44]^ also indicated elevation of HDL values, however, the relation between changes of HDL and weight loss were not found.

Analysis of TG values, allows us to state that LSG decreases TG concentration in the blood. The results were statistically significant. Zhang et al^[40]^ indicate similar conclusion; they also point out the fact that 22.2% of patients after LSG still need a pharmacotherapy.

Nowadays, it is considered that fat tissue is responsible for homeostasis and plays an important role in human metabolism. Furthermore, adipose tissue macrophages, which are a source of pro- and antiinflammatory cytokines, seem to be relevant in development of insulin resistance. Correlation between CRP and BMI in obesity is well known. It was proved that weight loss results in CRP decrease.^[45,46]^ In our study, average CRP level was between the laboratory reference rate in every step of the follow-up. Only in studied Group II and III reduction of CRP, 6 months after the LSG, was statistically significant. Wong et al^[44]^ observed statistically significant reduction of CRP connected with weight loss after LSG. Iannelli et al^[39]^ stated significant correlation between CRP and development of metabolic syndrome.

The most serious complications of LSG are staple line leaks and bleeding, and occur in 1% to 3% of patients.^[47–49]^ Other complications include biliary complications, for example. acute pancreatitis which may occur in 9.4%, but also, stenosis, abdominal abscess, pulmonary embolism, deep venous thrombosis may appear.^[47,48,50,51]^ However, according to the position statement on sleeve gastrectomy as a bariatric procedure by American Society for Metabolic and Bariatric Surgery (ASMBS), LSG is a preferred method of obesity treatment as a first-step management.^[52]^ In our study the complication rate was 2.5% (n = 3). We have observed acute pancreatitis, bleeding from the first trocar site into the abdominal cavity and superficial thrombophlebitis of the lower extremity. All complications concerned female, who did not follow tobacco smoking restrictions in the postoperative period. The acute pancreatitis was successfully treated by fluid resuscitation, pain control, and nutritional support. To control the bleeding, the incision line was enlarged, the bleeding vessel was identified and coagulated. A venous inflammation was limited to the one superficial vein and it was less than 5 cm, thus conservative treatment was applied and the symptoms disappeared in the next few days.

There were several limitations in the study. First of all, our study had a 120 patients sample size, which could potentially affect the accuracy of the analysis. Secondly, all patients were Caucasian and came from the one region of Poland, however the results were comparable with the worldwide literature. Finally, due to a very limited group of patients who underwent a follow-up examination after a year, a long-term effect of LSG is still unclear.

Collection of long-term data is warranted and analysis of multicenter data should be conducted.

## 5 Conclusion

According to our results, LSG is effective method of obesity treatment. Body mass loss expressed in %EWL and %EBMIL is the most noticeable when a small bougie size is used. We have shown that LSG significantly improves carbohydrates and lipids parameters, even before a noticeable weight loss is observed. In our opinion there is still a need for further studies on inflammatory factors and liver enzymes levels in obesity. Observation of these parameters will allow to find a connection between obesity and development of comorbidities and metabolic syndrome.

## References

[1] Obesity and overweight Fact sheet N°311. WHO. January 2015. Retrieved 2 February 2016.

[2] Bastard JP Maachi M Lagathu C Recent advances in relationship between obesity, inflammation and insulin resistance. Eur Cytokine Netw 2006;17:4–12. 16613757

[3] Pucci G Alcidi R Tap L Sex- and gender-related prevalence, cardiovascular risk and therapeutic approach in metabolic syndrome: A review of the literature. Pharmacol Res 2017;120:34–42. 28300617 10.1016/j.phrs.2017.03.008

[4] Milic S Lulic D Stimac D Non-alcoholic fatty liver disease and obesity: biochemical, metabolic and clinical presentations. World J Gastroenterol 2014;20:9330–7. 25071327 10.3748/wjg.v20.i28.9330 PMC4110564

[5] Van der Bruggen M Bos G Bemelmans W Lifestyle interventions are cost-effective in people with different levels of diabetes risk. Results from a modeling study. Diabetes Care 2007;30:128–34. 17192345 10.2337/dc06-0690

[6] Amit G Naidu CS Rao PP The effect of Laparoscopic Sleeve Gastrectomy on glycemic control in morbidly obese patients. Int J Surg 2016;28:131–5. 26902533 10.1016/j.ijsu.2016.02.063

[7] Hady HR Dadan J Gołaszewski P Impact of laparoscopic sleeve gastrectomy on body mass index, ghrelin, insulin and lipid levels in 100 obese patients. Wideochir Inne Tech maloinwazyjne 2012;7:251–9. 23362424 10.5114/wiitm.2011.28979 PMC3557740

[8] Langer FB Reza Hoda MA Bohdjalian A Sleeve gastrectomy and gastric banding: effects on plasma ghrelin levels. Obes Surg 2005;15:1024–9. 16105401 10.1381/0960892054621125

[9] Hady HR Dadan J Luba M The influence of laparoscopic sleeve gastrectomy on metabolic syndrome parameters in obese patients in own material. Obes Surg 2012;22:13–22. 21986646 10.1007/s11695-011-0530-y PMC3257432

[10] Alberti KG Zimmet P Shaw J Metabolic syndrome—a new world-wide definition. A Consensus Statement from the International Diabetes Federation. Diabet Med 2006;23:469–80. 16681555 10.1111/j.1464-5491.2006.01858.x

[11] Parikh M Gagner M Heacock L Laparoscopic sleeve gastrectomy: does bougie size affect mean %EWL? Short-term outcomes. Surg Obes Relat Dis 2008;4:528–33. 18656834 10.1016/j.soard.2008.03.245

[12] Yuval JB Mintz Y Cohen MJ The effect of bougie caliber on leaks and excess weight loss following laparoscopic sleeve gastrectomy. Is there an ideal bougie size? Obes Surg 2013;23:1685–91. 23912264 10.1007/s11695-013-1047-3

[13] Weiner RA Weiner S Pomhoff I Laparoscopic sleeve gastrectomy – Influence of sleeve size and resected gastric volume. Obes Surg 2007;17:1297–305. 18098398 10.1007/s11695-007-9232-x

[14] Mongol P Chosidow D Marmuse J Laparoscopic sleeve gastrectomy as an initial bariatric operation for high risk patients: initial results in 10 patients. Obes Surg 2005;15:1030–3. 16105402 10.1381/0960892054621242

[15] Han S Kim W Oh J Results of laparoscopic sleeve gastrectomy (LSG) at 1 year In morbidly obese Korean patients. Obes Surg 2005;15:1469–75. 16354529 10.1381/096089205774859227

[16] Dixon JB Zimmet P ALberti KG Rubino F International Diabetes Federation Taskforce on Epidemiology and Prevention. Bariatric Surgery: an IDF statement for obese type 2 diabetes. Surg Obes relat Dis 2011;7:433–47. 21782137 10.1016/j.soard.2011.05.013

[17] Mokdad AH Ford ES Bowman BA Prevalence of obesity, diabetes and obesity-related high risk factors. JAMA 2001;289:76–9. 10.1001/jama.289.1.76 12503980

[18] Rizzello M Abbatini F Casella G Early postoperative insulin-resistance changes after sleeve gastrectomy. Obes Surg 2010;20:50–5. 19916040 10.1007/s11695-009-0017-2

[19] Catoi AF Parvu A Mironiuc A Effects of sleeve gastrectomy in insulin resistance. Clujul Med 2016;89:267–72. 27152079 10.15386/cjmed-576 PMC4849386

[20] Sharma R Hassan Ch Chaiban JT Severe insulin resistance improves immediately after sleeve gastrectomy. J Investig Med High Impact Case Rep 2016;4:1–3. 10.1177/2324709615625309 PMC4710130 26788532

[21] Hady HR Gołaszewski P Zbucki RL The influence of laparoscopic adjustable gastric banding and laparoscopic sleeve gastrectomy on weight loss, plasma ghrelin, insulin, glucose and lipids. Folia Histochem Cytobiol 2012;50:292–303. 22763970 10.5603/fhc.2012.0039

[22] Peterli R Steinert RE Woelnerhanssen B Metabolic and hormonal changes after laproscopic Roux-en-Y gastric bypass and sleeve gastrectomy: a randomized, prospective trial. Obes Surg 2012;22:740–8. 22354457 10.1007/s11695-012-0622-3 PMC3319900

[23] Karamanakos SN Vagenas K Kalfarentzos F Weight loss, appetite suppression and changes in fasting and postprandial ghrelin and peptide-YY levels after Roux-en-Y gastric bypass and sleeve gastrectomy: a prospective, double blind study. Ann Surg 2008;247:401–7. 18376181 10.1097/SLA.0b013e318156f012

[24] Guidone C Manco M Valera-Mora E Mechanisms of recovery from type 2 diabetes after malabsorptive bariatric surgery. Diabetes 2006;55:2025–31. 16804072 10.2337/db06-0068

[25] Briatore L Salani B Andraghetti G Restoration of acute insulin response in T2DM subjects 1 month after biliopancreatic diversion. Obesity 2008;16:77–81. 18223616 10.1038/oby.2007.9

[26] Rubino F Is type 2 diabetes an operable intestinal disease? A provocative yet reasonable hypothesis. Diabetes Care 2008;31 (Suppl 2):S290–6. 18227499 10.2337/dc08-s271

[27] Scott WR Batterham RL Roux-en-Y gastric bypass and laparoscopic sleeve gastrectomy: understanding weight loss and improvements in type 2 diabetes after bariatric surgery. Am J Physiol Regul Integr Comp Physiol 2011;301:R15–27. 21474429 10.1152/ajpregu.00038.2011

[28] Vigneshwaran B Wahal A Aggarwal S Impact of sleeve gastrectomy on type 2 diabetes mellitus, gastric emptying time, glucagon-like peptide 1 (GLP – 1), ghrelin and leptin in non-morbidly obese subjects with BMI 30 – 35 kg/m2: a prospective study. Obes Surg 2016;26:2817–23. 27185177 10.1007/s11695-016-2226-9

[29] Basso N Capoccia D Rizzello M First-phase insulin secretion, insulin sensitivity, ghrelin, GLP-1 and PYY changes after 72 h after sleeve gastrectomy in obese diabetis patients: the gastric hypothesis. Surg Endosc 2011;25:3540–50. 21638183 10.1007/s00464-011-1755-5

[30] Peterli R Woelnerhanssen B Peters T Improvement in glucose metabolism after bariatric surgery comparison of laparoscopic Roux-en-Y gastric bypass and laparoscopic sleeve gastrectomy: a randomized, prospective trial. Ann Surg 2009;250:234–41. 19638921 10.1097/SLA.0b013e3181ae32e3

[31] Ariyasu H Takaya K Tagami T Stomach is a major source of circulating ghrelin, and feeding state determines plasma ghrelin-like immunoreactivity levels in humans. J Clin Endocrinol Metab 2001;86:4753–8. 11600536 10.1210/jcem.86.10.7885

[32] Neeland IJ Turer AT Ayers CR Dysfunctional adiposity and the risk of prediabetes and type 2 diabetes in obese adults. Jama 2012;308:1150–9. 22990274 10.1001/2012.jama.11132 PMC3556508

[33] Almazeedi S Al-Sabah S Alshammari D Routine trans-abdominal ultrasonography before laparoscopic sleeve gastrectomy: the findings. Obes Surg 2014;24:397–9. 24101090 10.1007/s11695-013-1092-y

[34] Adibi A Kelishadi R Beihagi A Sonographic fatty liver in overweight and obese children, a cross sectional study in Isfahan. Endokrynol Pol 2009;60:14–9. 19224500

[35] Vajro P Lenta S Socha P Diagnosis of nonalcoholic fatty liver disease in children and adolescents: position paper of the ESPGHAN Hepatology Committee. J Pediatr Gastroenterol Nutr 2012;54:700–13. 22395188 10.1097/MPG.0b013e318252a13f

[36] Jaser N Mustonen H Pietila J Preoperative transabdominal ultrasonography (US) prior to laparoscopic Roux-en-Y gastric bypass (LRYGBP) an laparoscopic sleeve gastrectomy (LSG) in the first 100 operations. Was it beneficial and reliable during the learning curve? Obes Surg 2012;22:416–21. 21660643 10.1007/s11695-011-0416-z

[37] Karelis AD St-Pierre DH Conus F Metabolic and body composition factors in subgroups of obesity: what do we know? J Clin End and Metab 2004;89:2569–75. 10.1210/jc.2004-0165 15181025

[38] Vix M Diana M Liu KH Evolution of glycolipid profile after sleeve gastrectomy vs. Roux-en-Y Gastric bypass: results of a prospective randomized clinical trial. Obes Surg 2013;23:613–21. 23207829 10.1007/s11695-012-0827-5

[39] Iannelli A Anty R Schneck AS Inflammation, insulin resistance, lipid disturbances, anthropometrics and metabolic syndrome in morbidly obese patients: A case control study comparing laparoscopic Roux-enY gastric bypass and laparoscopic sleeve gastrectomy. Surgery 2011;149:264–70. 10.1016/j.surg.2010.08.013 20932542

[40] Zhang F Strain GW Lei W Changes in lipid profiles in morbidly obese patients laparoscopic sleeve gastrectomy. Obes Surg 2011;21:305–9. 20859769 10.1007/s11695-010-0285-x

[41] Gerard P Metabolsim of cholesterol and bile acids by the gut microbiota. Pathogens 2013;3:14–24. 25437605 10.3390/pathogens3010014 PMC4235735

[42] Li L Batt SM Wannemuehler M Effect of feeding of a cholesterol-reducing bacterium, Eubacterium coprostanoligenes, to germ-free mice. Lab Anim Sci 1998;48:253–5. 10090024

[43] El Khoury D Hwalla N Frochot V Postprandial metabolic and hormonal responses of obese dyslipidemic subjects with metabolic syndrome to test meals, rich in carbohydrate, fat or protein. Atherosclerosis 2010;210:307–13. 20031131 10.1016/j.atherosclerosis.2009.11.017

[44] Wong ATY Chan DC Armstrong J Effect of laparoscopic sleeve gastrectomy on elevated C-reactive protein and atherogenic dyslipidemia in morbidly obese patients. Clin Biochem 2011;44:342–4. 21167144 10.1016/j.clinbiochem.2010.12.004

[45] Pardina E Ferrer R Baena-Fustegeras JA Only C-reactive protein but not TNF-ɑ or IL6, reflects the improvement in inflammation after bariatric surgery. Obes Surg 2012;22:131–9. 22038572 10.1007/s11695-011-0546-3

[46] Frohlich M Imhof A Berg G Association between C-reactive protein and features of the metabolic syndrome: a population-based study. Diabetes Care 2000;23:1835–9. 11128362 10.2337/diacare.23.12.1835

[47] Basso N Casella G Rizzello M Laparoscopic sleeve gastrectomy as first stage or definitive intent in 300 consecutive cases. Surg Endosc 2011;25:444–9. 20607564 10.1007/s00464-010-1187-7

[48] Bellanger DE Greenway FL Laparoscopic sleeve gastrectomy, 529 cases without a leak: short-term results and technical considerations. Obes Surg 2011;21:146–50. 21132397 10.1007/s11695-010-0320-y

[49] Spyropoulos C Argentou MI Petsas T Management of gastrointestinal leaks after surgery for clinically severe obesity. Surg Obes Relat Dis 2011;21:1650–6. 10.1016/j.soard.2011.04.222 21616725

[50] Chang J Corcelles R Boules M Predictive factors of biliary complications after bariatric surgery. Surg Obes Relat Dis 2016;12:1706–10. 26948453 10.1016/j.soard.2015.11.004

[51] Javanainen M Penttilä A Mustonen H A Retrospective 2-Year Follow-up of Late Complications Treated Surgically and Endoscopically After Laparoscopic Roux-en-Y Gastric Bypass (LRYGB) and Laparoscopic Sleeve Gastrectomy (LSG) for Morbid Obesity. Obes Surg 2017;doi: 10.1007/s11695-017-2967-0. [Epub ahead of print]. 10.1007/s11695-017-2967-0 29080042

[52] ASMBS Clinical Issues Committee: Updated position statement on sleeve gastrectomy as a bariatric procedure. Surg Obes Relat Dis. 2012 May-Jun;8(3):e21-6. 10.1016/j.soard.2012.02.001 22417852

